# The Role of State Health Departments in Advancing a New Mental Health Agenda

**Published:** 2009-12-15

**Authors:** Geraldine S. Perry, Rachel Guerrero, Doreleena Sammons-Posey, Valerie J. Edwards, Sharrice White-Cooper, Letitia Presley-Cantrell

**Affiliations:** Centers for Disease Control and Prevention; California Department of Mental Health, Sacramento, California; National Association of Chronic Disease Directors and Directors of Health Promotion and Education, Trenton, New Jersey; Centers for Disease Control and Prevention, Atlanta, Georgia; Centers for Disease Control and Prevention, Atlanta, Georgia; Centers for Disease Control and Prevention, Atlanta, Georgia

Throughout the 20th century, the mission of local and state public health departments broadened from infectious disease prevention and control to encompass maternal and child health, immunizations, food and water safety, environmental health, and chronic disease prevention (1-3). Now it is recognized that mental illness, especially depression, influences the treatment and outcomes of many chronic diseases (4). However, mental health has traditionally been managed as a separate state and local service  focused on treating mental illnesses and substance abuse, not on preventing mental illness and promoting mental health. Few state public health departments have integrated physical and mental health services.

The Institute of Medicine has recommended that "each state have a department of health that groups all primarily health-related functions" (5). It further suggests that these health departments be responsible for the prevention of physical and mental illness, the promotion of physical and mental health, and the prevention and treatment of substance abuse. The World Health Organization has stated that, "Mental health — neglected for far too long — is crucial to the overall well-being of individuals, societies, and countries and must be universally regarded in a new light" (6). In a survey of 30 state health departments conducted by the Directors of Health Promotion and Education, only 6 reported that their health departments had policies that included mental health in their health promotion and health education efforts (written communication, Directors of Health Promotion and Education, 2009). Twelve said they were considering an integrated model, 10 were already working to incorporate these issues, and 4 wanted more information. Only 1 of the state health departments surveyed already had integrated physical and mental health services.

Nineteen respondents believed that mental health issues fit best under "community health" on the public health agenda, but all the respondents acknowledged that public health professionals should promote mental health in the context of public health issues. Although the survey indicates that states want their public health models to encompass mental health, funding and administrative barriers exist. Federal funding for state mental health services is separate from that for state public health departments and provides little support for prevention services. States have few incentives to integrate physical and mental public health care. Funding for mental illness prevention comes from limited state and county resources. These factors discourage integration of services and do not recognize the vital role that mental health plays in chronic disease outcomes.

In 2004, California voters passed Proposition 63 (Mental Health Services Act — Welfare and Institutions Code 5890), which supports the integration of primary care and mental health. The purposes of this law were to 1) define serious mental illness as a condition deserving priority attention, including prevention and early-intervention services and medical and supportive care; 2) reduce the long-term adverse effect for people and state and local budgets resulting from untreated serious mental illness; 3) expand successful, innovative service programs (including those that integrate physical and mental health); 4) make state funds available to provide services that are not already covered by federally sponsored programs; and 5) develop services that are best practices and subject to local and state oversight. Proposition 63 finances county-based mental health programs to expand overall services, including prevention and early-intervention programs. These investments will support the growth of capacity for more integration of mental health and primary care, thus establishing new practice models that will be worth watching. The California Mental Health Directors Association has reported that several counties are using their Proposition 63 dollars to fund integration of mental health and primary care programs. For example, San Diego County has established new mental health services in a primary care diabetes clinic that serves a large Latino population to treat and prevent depression and diabetes in an integrated approach.

To support integration, state and local health departments and mental health departments must take a more active role in developing specific strategies and identifying system-level support to begin working together. For example, pilot projects could be developed that use integrative approaches to encourage local mental health providers to collaborate with public health providers and increase access to mental health treatment for racial and ethnic minorities. Such efforts could address disparities among minorities who are not accessing mental health treatment. Health departments could also better inform the public of the role that mental health plays in overall prevention and treatment of many chronic diseases. Both public health and mental health providers need training to increase their understanding that there is no health without mental health. Furthermore, public health and mental health professionals should work together to convince funders that integration of public health and mental health is important to overall health. Through this effort, national legislation, policies, and funding could begin to provide incentives for public health and mental health integration.
